# Multidimensional Analysis of Parent-Perceived Quality of Life in Children with Cerebral Palsy: A Cross-Sectional Study

**DOI:** 10.3390/children13010128

**Published:** 2026-01-15

**Authors:** Javier López-Ruiz, María-José Giménez, Marina Castel-Sánchez, Patricia Rico-Mena, Ana Mallo-López, Federico Salniccia, Patricia Martín-Casas

**Affiliations:** 1 Universidad Europea de Madrid, Faculty of Medicine, Health and Sports, Department of Physiotherapy, Campus de Villaviciosa, Calle Tajo s/n, 28670 Villaviciosa de Odón, Madrid, Spain; javier.lopez3@universidadeuropea.es (J.L.-R.); marina.castel@universidadeuropea.es (M.C.-S.); patricia.rico@universidadeuropea.es (P.R.-M.); ana.mallo@universidadeuropea.es (A.M.-L.); federico.salniccia@universidadeuropea.es (F.S.); 2 Department of Physiotherapy, Faculty of Nursing, Physiotherapy and Podiatry, Complutense University of Madrid, Plaza Ramón y Cajal, 3, 28040 Madrid, Spain; pmcasas@ucm.es; 3 InPhysio Research Group, Health Research Institute of the Hospital Clínico San Carlos (IdISSC), 28040 Madrid, Spain

**Keywords:** cerebral palsy, children, health-related quality of life, parents, international classification of functioning, disability and health

## Abstract

**Highlights:**

**What are the main findings?**

• Parents of children using AFOs, receiving botulinum toxin, undergoing pelvic surgery, or engaging in intensive physical therapy (>2 h/week) reported significantly lower scores in multiple domains of quality of life, including “Social well-being”, “Emotional well-being”, “Feelings about functioning”, and “Family health”.
• The ‘Feelings about Functioning’ domain was 62% explained by motor and activity-related measures (GMFM-88, PEDI-CAT Activity, and PEDI-CAT Social/Cognitive), with PEDI-CAT Activity emerging as the strongest predictor.

**What are the implications of the main findings?**

• The association between specific medical interventions and lower quality-of-life scores underscores the need for holistic care strategies that address psychosocial well-being alongside physical treatment in children with CP.
• Identifying PEDI-CAT Activity as the strongest predictor of the ‘Feelings about Functioning’ domain highlights the importance of prioritizing functional activity in rehabilitation programs to enhance perceived quality of life.

**Abstract:**

**Background/Objectives**: To analyze the parent-perceived quality of life (QoL) in children with cerebral palsy (CP) and to study the relationship between sociodemographic and clinical factors and this perception, under the perspective of the International Classification of Functioning, Disability and Health (ICF). **Methods**: A cross-sectional study was conducted with 95 participants (ages 5–19 years) with CP. Participants’ parents were asked about sociodemographic and clinical characteristics and compiled Cerebral Palsy Quality of Life (CP-QoL) and Pediatric Disability Inventory-Computer Adaptive Test (PEDI-CAT). Participants were assessed and classified into the following functional domains: gross motor function (GMFM-88, GMFCS), manual ability (MACS), eating and drinking abilities (EDACS), and communication function (CFCS). Correlations between CP-QoL domains and variables were investigated using Spearman’s correlation coefficient and multivariate predictive models were used to investigate the variables predicting CP-QoL scores for each domain. **Results**: A total of 95 children with a mean age of 12.4 ± 3.5 years (range 5–19 years) were included. Participants demonstrated moderate-high GMFM-88 level (228.8 ± 44.7) and high functional performance across PEDI-CAT domains: Activity (57.2 ± 5.1), Mobility (63.1 ± 5.6), and Social/Cognitive (70.2 ± 4.3). Parent-perceived QoL was significantly higher when children did not require AFOs, botulinum toxin, or recent hospitalizations, and lower among children who attended physical therapy >2 h/week. Moderate correlations were consistently found between the ‘Feelings about Functioning’ domain and functional variables, being positive for GMFM-88 and all PEDI-CAT domains, and negative for GMFCS, MACS, EDACS and CFCS. That domain of CP-QoL was best explained by the regression model (R^2^ = 0.619, *p* < 0.001), with the combination of three variables: GMFM-88, PEDI-CAT Activity and PEDI-CAT Social/Cognitive. Among them, PEDI-CAT Activity was the strongest predictor (β = 0.1436). **Conclusions:** In children with CP, to enhance family well-being, interventions should prioritize social participation and carefully balance the intensity and frequency of therapy against family burden and daily life demands, as QoL is primarily driven by manual ability and functional performance.

## 1. Introduction

Cerebral Palsy (CP) encompasses a diverse range of developmental disorders affecting movement and posture, leading to activity limitations. These deficits result from non-progressive brain lesions occurring during the perinatal period [1]. CP represents the leading cause of significant motor dysfunction and physical disability in children, with a prevalence of approximately 1.6 per 1000 live births in high-income regions, including Europe and the United States [2]. Children with CP often experience a reduced quality of life (QoL), mainly due to neuromusculoskeletal impairments, activity restrictions, and participation restrictions [3]. Traditionally, children with cerebral palsy engage in ongoing, multidisciplinary rehabilitation across childhood and adolescence. In most settings, this entails regular attendance, often multiple sessions per week, at pediatric rehabilitation services.

The World Health Organization Quality of Life Assessment Group defines QoL as ‘an individual’s subjective perception of their satisfaction across various domains in life in the context of the culture and value systems in which they live and in relation to their goals, expectations, standards and concerns’ [4]. However, QoL in pediatric populations captures the multifaceted effects of health conditions and their treatments across physical, psychological, social, and behavioral dimensions. It serves as a valuable tool for providing a holistic overview of a child’s well-being, enabling the identification of both positive and negative aspects, monitoring changes over time, and evaluating responses to treatment [5]. In children with CP, QoL assessments have predominantly been used to examine the effects of various interventions [6]. Specific instruments, such as the Pediatric Quality of Life Inventory (PedsQL) [7], the KIDSCREEN [8] and the Cerebral Palsy Quality of Life (CP-QoL) questionnaire [9,10,11], have been developed to cater to this population.

Given the complexity of assessing QoL in children, together with the breadth of the existing theoretical frameworks and the difficulty of applying them in clinical practice, previous research has explored the applicability of the International Classification of Functioning, Disability, and Health (ICF) [12] model in this context. The ICF provides a structured approach to understanding the complex interplay among body functions and structures, activities and participation, and personal and environmental factors, all of which contribute equally to health and functionality. As children grow and develop, each of these components can influence and shape their QoL [12]. Some evidence suggests that psychosocial and emotional well-being is not necessarily directly related to functional status, defined in terms of body function, structure, activity, and participation [13,14]. However, the cultural differences between countries and regions and the specificities of each child with CP and their context (family, school, environment) call for more studies that specifically assess how different factors may be barriers or facilitators to children’s development and QoL.

Despite limitations in physical function, children with CP may experience positive psychosocial and emotional well-being [14]. Consequently, QoL assessments should prioritize psychosocial factors and subjective experiences rather than focusing solely on functional impairments or symptoms [15,16]. As QoL is inherently subjective, it is ideally self-reported by individuals [15]. However, certain pediatric populations, particularly children younger than eight years, as well as those presenting with developmental delays, severe functional limitations, cognitive deficits, or significant communication disorders, may lack the capacity to complete self-reported QoL assessments reliably. In such instances, proxy reports from parents or caregivers serve as valuable alternatives [15,17].

The use of QoL instruments as outcome measures in research and clinical practice has increased, particularly in assessing psychosocial and emotional well-being to evaluate the effectiveness of interventions for children with CP [5,15]. However, most assessment efforts have traditionally focused on body function and activities, particularly in rehabilitation contexts, as children with CP often show motor impairments such as deficits in balance and postural control during functional tasks [18], as well as gait disturbances [19]. Consequently, assessment in children with CP has relied mainly on instruments focused on motor performance and functional classification, such as the Gross Motor Function Measure–88 (GMFM-88) [20,21] and classification systems including Gross Motor Function Classification System (GMFCS) [22], the Manual Ability Classification System (MACS) [23], the Communication Function Classification System (CFCS) [24], and the Eating and Drinking Ability Classification System (EDACS) [25]. Although these tools provide valuable information on body functions and activity limitations, they offer limited insight into how such impairments translate into autonomy and participation in everyday contexts [26]. To address this gap, instruments such as the Pediatric Evaluation of Disability Inventory–Computer Adaptive Test (PEDI-CAT) have been increasingly used to assess performance in daily activities, mobility, social/cognitive domains, and responsibility [27].

However, all these tools primarily focus on children’s performance and do not sufficiently address the influence of environmental factors, as recommended by ICF [28] and the latest clinical practice guideline for the management of children and young people with CP [29]. As parents are the main caregivers, particularly for younger, more severely affected or non-communicative children, their perspective is essential to capture participation restrictions and contextual barriers affecting everyday activities.

Therefore, the primary objective of this study was to analyze parent-perceived QoL in children with CP. A secondary aim was to examine the relationships between sociodemographic and clinical factors and the QoL domains, identifying potential predictors through multivariate regression models.

## 2. Materials and Methods

### 2.1. Study Design

This observational cross-sectional study was carried out within the framework of a broad observational project conducted at the Hospital Infantil Universitario Niño Jesús, a major public pediatric hospital in Madrid, Spain. The project comprised several interrelated studies in children with CP to address different research objectives. A total sample of 105 children was initially recruited for the overall project. Two of the studies derived from this project have been previously published [30,31]. For the present analysis, a subsample of 95 children with CP was selected from the original cohort according to the specific study aims and inclusion criteria that required a confirmed diagnosis of cerebral palsy (CP), age between 5 and 19 years, ability to sit unsupported, and ability to follow test instructions. In addition, participants should not have undergone orthopedic surgery or botulinum toxin injections in the six months prior to the study.

The study was approved by the ethics committee of the Bio-Medical Foundation of the Hospital Infantil Universitario Niño Jesús of Madrid (registration number: R-0066/20).The study adhered to the ethical principles of the Declaration of Helsinki (World Medical Association, 2013) and followed the Strengthening the Reporting of Observational Studies in Epidemiology (STROBE) [32] statement. Written informed consent was obtained from individuals 12 years of age or older before study participation, and verbal consent was obtained from younger participants, with parental written consent obtained in all cases. Both children and parents confirmed their willingness to participate. The sample size aligned with or exceeded that of published studies with a similar objective [33,34].

### 2.2. Outcomes

#### 2.2.1. Cerebral Palsy Quality of Life (CP-QoL)

The Cerebral Palsy Quality of Life (CP-QoL) questionnaire has been specifically designed to assess the quality of life of children with CP, with versions tailored for parents/caregivers and for children and adolescents [9]. It uses a 9-point rating scale to measure perceived well-being (ranging from 1 = very unhappy to 9 = very happy), with scores subsequently converted to a 0–100 scale. The CP-QoL has demonstrated strong psychometric properties in previous studies [6,35].

The Spanish version [11] for parents/caregivers consists of a 74-item scale across seven domains: “Social Well-being, Acceptance, and Participation”; “Feelings about Functioning”; “Emotional Well-being and Self-Esteem”; “Pain and Impact of Disability”; “School”; “Access to services”; and “Family health”. This instrument is designed for parents of children aged 4 to 18 years.

#### 2.2.2. Pediatric Disability Inventory Computer Adaptive Test (PEDI-CAT)

The PEDI-CAT assesses a child’s ability and performance in activities of daily living. It consists of a bank of 267 items divided into four independent domains: Activities of Daily Living, Mobility and Social/Cognitive [36]. The instrument is designed for use from birth to 21 years of age and can be administered by parents or professionals using a computer or tablet. The estimated time to complete each domain is approximately 13 min [37]. A Spanish version of the PEDI-CAT (version 1.4.3, January 2019) was available through Pearson [38].

#### 2.2.3. Gross Motor Function Measure-88 (GMFM-88)

The GMFM-88 assesses gross motor function by quantifying the movements a child can and cannot perform. However, it does not evaluate the quality of movements [39,40]. It is considered the gold standard for measuring gross motor function in pediatric neurological disorders and has been validated in Spanish population with CP [41]. The assessment consists of 88 items divided into five dimensions: (A) lying and rolling, (B) sitting, (C) crawling and kneeling, (D) standing, and (E) walking, running, and jumping. Each item is scored based on the best performance from three attempts, with results expressed as percentages. Although administration time varies, it is generally reported to take less than 60 min [39].

#### 2.2.4. Gross Motor Function Classification System (GMFCS)

The GMFCS categorizes gross motor function in children with CP into five levels based on self-initiated movement and functional limitations, focusing on usual performance in everyday settings [42,43]. Levels I–III range from independent walking to the need for assistive devices, while Levels IV–V describe severely limited mobility requiring transportation or powered mobility. The system shows adequate content validity and moderate-to-good inter-rater reliability (ICC = 0.75 for ages 2–12). In 2007, the expanded and revised version (GMFCS-E&R) was introduced to assess children and adolescents aged 12 to 18 years [22].

#### 2.2.5. Manual Ability Classification System (MACS)

The MACS categorizes how children with CP handle objects in daily activities, focusing on typical bimanual performance rather than maximal capacity [23]. It comprises five levels based on autonomy and quality of object handling, considering the need for assistance or adaptations. Reliability is excellent, with inter-rater ICC = 0.97 and parent-professional agreement ICC = 0.96 [44].

#### 2.2.6. Communication Function Classification System (CFCS)

The CFCS is a five-level scale for classifying everyday communication effectiveness in individuals with cerebral palsy, categorizing performance based on sender/receiver roles, pace, and interaction partner familiarity. It demonstrated strong psychometric properties, with good content validity, professional inter-rater reliability (ICC ≈ 0.66–0.77), and excellent test–retest reliability (ICC ≈ 0.82) in a validation sample of 2–18-year-olds with CP [24].

#### 2.2.7. Eating and Drinking Ability Classification System (EDACS)

The EDACS, comprising five levels, evaluates the safety and efficiency of eating and drinking in this population. It exhibited excellent inter-rater reliability (ICC ≥ 0.94) and strong concurrent validity, correlating highly with established dysphagia and functional measures such as the Bogenhausener Dysphagiescore, MACS, and WeeFIM eating items [25].

### 2.3. Study Procedures

After confirmation of the inclusion criteria, all relevant sociodemographic and clinical data were collected: parent’s educational level (primary/secondary, intermediate, university), parent’s occupation (unemployed, employed, separated, care allowance), diagnostic (hemiparesis, spastic diplegic CP, spastic quadriplegic CP, ataxic CP), significant cognitive or language disability (yes, no), previous (>6 months) surgery affecting pelvis and/or botulinum toxin (yes, no), need of ankle–foot orthoses (AFO) (none, daytime AFO, nighttime AFO, daytime AFO + nighttime AFO), assistive devices (none, walker, wheelchair, walker + wheelchair), glasses (yes, no), standing frame (yes, no), and attendance to school (special, ordinary with adaptations, ordinary). Therapies received were also recorded, including physical therapy (none or <1 h/week, 1–2 h/week, >2–3 h/week, and >3 h/week), occupational therapy (<2 h/week, >2 h/week), and speech therapy (<2 h/week, >2 h/week). Dichotomous variables were created for Physical therapy (<2 h/week, >2 h/week) and the need of ankle–foot orthoses (AFO) (yes, no). All in-person assessments were then performed on the same day. A single researcher administered the GMFM-88. The total assessment time was estimated to be between 90 and 120 min. During the assessments, parents completed the PEDI-CAT and the CP-QoL questionnaires. Another researcher was available to address any questions or concerns that arose.

All data were systematically recorded on an evaluation sheet and reviewed before the assessment. Any uncertainties among participants or their parents were addressed, and the assessment results were communicated.

All procedures complied with the regulations on the protection of personal data. The collection, processing, communication, and transfer of patient data complied with the requirements of the Spanish law on protection of personal data and guarantee of digital rights, and European general data protection regulation.

### 2.4. Data Analysis

Data analyses were performed using Jamovi (Version 2.6.44; The Jamovi Project, 2024). The normality of the variables was assessed using the Kolmogorov–Smirnov test and Q-Q plots. Given the non-normal distribution of the variables, the median (P25, P75) was used to described quantitative variables, and non-parametric tests were applied. The Mann–Whitney U test or the Kruskal–Wallis test were used for comparisons between groups. Correlations between CP-QoL domains and variables were investigated using Spearman’s correlation coefficient. Correlation coefficients were classified as negligible (ρ < 0.3), low (ρ ≥ 0.3 and <0.5), moderate (ρ ≥ 0.5 and <0.7), high (ρ ≥ 0.7 and <0.9), and very high (ρ ≥ 0.9) [45].

Multivariate predictive models were used to identify variables predicting CP-QoL scores within each domain. A backward elimination approach was applied, including only variables that had previously shown a significant correlation with the respective domain. Adjusted R^2^ values were considered to assess model fit. Only models with no collinearity among predictors and normally distributed residuals were considered, thereby confirming the regression model assumption.

The level of significance was set at *p* < 0.05.

## 3. Results

A total of 95 participants with a mean age of 12.4 ± 3.5 years (range 5–19 years) were included. Fifty-one (53.7%) of them were males. Table 1 shows the distribution by age group, diagnosis and sociodemographic, and therapeutic characteristics of the participants. As shown, the age distribution was balanced, with a comparable representation of children and adolescents in the study. Hemiparesis and spastic diplegic CP were the most frequent diagnoses. Most participants used ankle–foot orthoses, mainly during daytime, while the majority did not require assistive mobility devices. More than 67% of the children attended ordinary school with adaptations, and 50.5% received more than 2 h per week of physical therapy.

Participants demonstrated moderate-high gross motor function (GMFM-88: 228.8 ± 44.7) and high functional performance across PEDI-CAT domains: Activity (57.2 ± 5.1), Mobility (63.1 ± 5.6), and Social/Cognitive (70.2 ± 4.3). Table 2 shows the functional classification of participants across different scales. Most children exhibited mild functional impairment, as few were classified at levels III or IV in the GMFCS (21.1%), MACS (21.1%), EDACS (9.5%), and CFCS (10.5%).

Table 3 presents differences in four CP-QoL domains—Social Well-being, Acceptance and Participation; Feelings about Functioning; Emotional Well-being and Self-esteem; and Family Health—according to dichotomous variables.

Participants using AFOs showed significantly lower scores in “Social well-being”, “Feelings about functioning”, and “Family health”. Those receiving botulinum toxin had significantly lower scores in “Social well-being”, “Emotional well-being”, and “Family health”. Participants who had undergone pelvic surgery reported significantly lower “Emotional well-being” and “Family health” scores. Significantly lower scores in “Feelings about functioning”, “Emotional well-being”, and “Family health” were reported by parents of children receiving more than 2 h per week of physical therapy. In addition, further differences were observed in the CP-QoL “Feelings about Functioning” domain according to walker/wheelchair use (yes/no: 10.8 ± 1.7 vs. 13.0 ± 1.7; *p* < 0.001), speech therapy (<2 vs. >2 h/week: 10.6 ± 1.7 vs. 12.7 ± 1.8; *p* < 0.001), diagnosis (Hemiparesis 12.8 ± 1.6 vs. Spastic diplegic CP 13.1 ± 1.7 vs. Spastic quadriplegic CP 10.9 ± 2.0 vs. Ataxic CP 10.5 ± 2.0; *p* < 0.001), and mother’s occupation (Unemployed 13.1 ± 1.5 vs. Employed 12.5 ± 2.0 vs. Separated 10.0 ± 0.0 vs. Care allowance 10.1 ± 1.5; *p* = 0.006). Differences were also found in the “Access to Services” domain according to diagnosis (Hemiparesis 12.0 ± 2.1 vs. Spastic diplegic CP 11.4 ± 2.5 vs. Spastic quadriplegic CP 10.5 ± 2.0 vs. Ataxic CP 9.0 ± 1.9; *p* = 0.007), and in “Emotional Well-being and Self-Esteem” according to father’s occupation (Unemployed 11.0 ± 0.0 vs. Employed 13.2 ± 2.2 vs. Separated 15.3 ± 1.0; *p* = 0.038).

Table 4 shows correlations between the seven CP-QoL domains and functional-related variables. As shown in the table, no significant correlations were found between the “Pain and Impact of Disability” domain and the variables tested whereas most significant correlations were related to the “Social Well-being, Acceptance, and Participation” and the “Feelings about Functioning” domains of CP-QoL.

Significant moderate correlations were consistently observed between the ‘Feelings about Functioning’ domain and functional variables; specifically, it correlated positively with functional performance measures and negatively with functional classification levels. Specifically, a strong positive correlation was found between ‘Feelings about Functioning’ and PEDI-CAT activity. Furthermore, moderate positive correlations were observed between this domain and Total GMFM-88, PEDI-CAT mobility, and PEDI-CAT social/cognitive. In contrast, significant moderate negative correlations were found between this domain and GMFCS, MACS, and the use of a walker or wheelchair.

Table 5 shows the predictive models developed using multiple linear regression analyses with all CP-QoL domains except the “Pain and Impact of Disability” and the “School” domains that had shown none or few correlated variables.

The “Feelings about Functioning” domain was the best explained in the regression model (R^2^ = 0.619, *p* < 0.001), with three variables (Total GMFM-88, PEDI-CAT Activity and PEDI-CAT Social/Cognitive) accounting for nearly 62% of the variance in this CP-QoL domain scores. Among these, PEDI-CAT Activity was the strongest predictor (β = 0.1436). The regression models for the “Social Well-being, Acceptance, and Participation” and “Emotional Well-being and Self-Esteem” domains were less predictive, with R^2^ values of 0.191 and 0.151, respectively. MACS emerged as a significant predictor for both domains, with negative β coefficients, indicating that greater manual impairment (higher MACS levels) was associated with lower CP-QoL scores. These findings suggest that reduced hand function may adversely affect social participation, acceptance, and emotional well-being in children with cerebral palsy. Interestingly, in the “Emotional Well-being and Self-Esteem” domain, physical therapy emerged as a negative predictor (β = −0.469).

## 4. Discussion

The present study aimed to analyze parent perception of QoL in children and adolescents with CP and its relationship with functional and clinical factors. Our findings suggest that parent perception of QoL is a multidimensional construct influenced not only by the child’s gross motor function, but also by clinical and functional factors. Clinical interventions, therapy frequency, and the use of orthopedic devices were associated with lower QoL. Functional performance and manual ability emerged as the strongest positive predictors.

Regarding participants’ characteristics, the sample had a mean age of 12.4 years and a distribution that predominantly included milder functional levels (GMFCS I and II) with a clinical profile characterized by hemiparesis and spastic diplegia. QoL in the CP population has been investigated in similar cohort [33], although those authors utilized the KIDSCREEN-27 instrument instead of the CP-QoL. Nevertheless, it is important to note that the Spanish versions of both scales have demonstrated a significant positive correlation, which may facilitate a reliable comparison of findings between these instruments [11].

Regarding functional status, participants exhibited a profile characterized by predominantly mild impairments across the major classification systems. The high representation of children at levels I and II of the GMFCS, MACS, EDACS, and CFCS is consistent with the robust scores achieved in the GMFM-88 and the various PEDI-CAT domains, particularly the Social/Cognitive dimension. This high-functioning profile suggests a sample with a considerable degree of independence in daily activities and social participation, the most frequent profile in studies of this population [46,47,48].

Perceived QoL among parents was significantly higher when their children did not require AFOs, botulinum toxin injections, surgery or recent hospitalizations. This suggests that ‘medicalization’ and clinical complexity negatively impact family well-being [34,49]. Socioeconomic factors such as income and maternal education are also important, but family income was not analyzed in the present study. These findings align with recent studies that emphasize the importance of a comprehensive clinical approach that integrates both medical stressors and the family’s socioeconomic context [34,50]. However, our results could be influenced because of most of the study participants used orthotic devices to walk and attended inclusive schools with specialized support. These environments provide the best context for the development of children with functional diversity. Supportive environments and social participation are associated with higher scores across several domains. The lack of income data used to classify the family’s socioeconomic status, as well as the lack of data on parental stress and environmental helpfulness, are limitations of the present study. These factors have been shown to impact CP-QoL scores significantly [51,52,53,54].

In other studies, the use of orthotic devices (AFOs) and wheelchairs has been associated with lower CP-QoL scores in the physical and participation domains, reflecting greater disability, but this does not necessarily predict lower scores in the psychosocial domains [55,56]. In contrast, our results have found an impact on all domains of CP-QoL: “Social Well-being, Acceptance, and Participation”, “Feelings about Functioning”, “Emotional Wellbeing and Self-esteem” and “Family Health”. Our results also highlight that a decrease in QoL was associated with a greater number of therapy hours per week. In fact, more than two hours of therapy per week may requires families to take the child to multiple sessions across the week. However, this relationship has not been established in the current literature, which has instead found that a longer duration of comprehensive rehabilitation (>6 months) may be associated with improved QoL [57].

A notable finding was the absence of significant correlations between the “Pain and Impact of Disability” domain and the functional variables assessed. This suggests that pain in children with CP is a complex and subjective experience that may not scale linearly with gross motor or manual impairment levels. These results align with previous research suggesting that pain is often influenced by individual comorbidities, psychological factors, or specific clinical interventions rather than global functional classifications [58,59]. In contrast, the “Social Well-being, Acceptance, and Participation” and “Feelings about Functioning” domains showed the strongest associations with functional status. This is consistent with longitudinal studies that identify functional performance as a primary driver of social integration and the child’s perceived competence in daily activities [34,59].

Notably, the “Feelings about Functioning” domain exhibited consistent moderate correlations with all functional metrics, demonstrating a positive relationship with performance-based measures while showing a negative association with functional classification levels. This dual correlation pattern suggests that parental satisfaction regarding their child’s competence is profoundly influenced by the interplay between the child’s clinical impairment (capacity) and their actual execution of daily life activities (performance), reflecting the importance of functional independence as a core component of perceived well-being [13,14,58].

Our results are like those of other studies that have found positive correlations between CP-QoL domains and clinical measures, such as the GMFM and PEDI-CAT scores, particularly in domains related to physical health, participation, and feelings about functioning [56,60]. However, whereas other authors have found that higher GMFM and PEDI-CAT scores are associated with a better QoL in the physical and participation domains, with the strongest correlation in the physical and functional domains and weaker associations in the psychosocial domains [60,61], in our sample, all CP-QoL domains are directly impacted by functional impairment, specially the ‘Feelings about Functioning’ domain, as previously discussed.

Stepwise regression identified manual ability (MACS) as the most consistent predictor for social, emotional, and family-related QoL domains. These results suggest that manual independence significantly influences social integration and emotional health, often surpassing the impact of gross motor function. This is consistent with evidence indicating that functional status is a primary determinant of well-being in the CP population [34] and underscores the complex relationship between activity limitations and the multifaceted nature of parent perceived QoL [13,50].

Regarding the “Feelings about Functioning” model it was the most robust (R^2^ = 0.409), with PEDI-CAT Activity and age as the primary predictors. This indicates that as children with CP mature, their perceived competence—and that of their parents—is increasingly tied to their functional performance in real-world settings, highlighting the transition from purely clinical capacity to daily activity as a key driver of well-being [59,62].

Finally, for “Emotional Well-being and Self-esteem”, MACS level and high therapy frequency (>2 h/week) emerged as the primary predictors (R^2^ = 0.334). Notably, higher therapy frequency functioned as a negative predictor, supporting the ‘treatment burden’ hypothesis; such high frequency, medicalized schedules likely curtail unstructured leisure and increase stress within the family unit, ultimately impacting the child’s emotional health [35].

These results are also consistent with the current literature, which affirms that the domains of the CP-QoL questionnaire show moderate to strong correlations with sociodemographic and clinical variables in children with CP, particularly regarding motor function, cognitive abilities, and environmental supports. Other studies have demonstrated that lower gross motor function (higher GMFCS level), poorer manual ability (MACS), reduced communication (CFCS), and eating/drinking ability (EDACS) are each independently associated with lower CP-QoL scores across most domains [57,61]. However, some authors have found that psychosocial domains (e.g., social and emotional wellbeing) may remain relatively intact even in children with severe motor impairment [61]. Our results revealed a significant impact of functional capacity on the ‘Feelings about Functioning’ and ‘Emotional Well-being and Self-esteem’ domains. The specific characteristics of the sample may influence this and should be examined in future studies that include more variables, such as data on family socioeconomic status, parental stress and environmental helpfulness.

### 4.1. Limitations

This study has some limitations that should be considered when interpreting the results. First, quality of life was assessed solely through parent-proxy reports. While self-reporting is generally considered the “gold standard”, proxy questionnaires are recognized as the most appropriate and reliable option for evaluating QoL in children under eight years of age or those with significant cognitive and communicative challenges [10,17]. Second, the sample was predominantly composed of children with mild functional impairments (GMFCS levels I and II). As this sample was primarily selected to validate the Trunk Control Measure Scale [30], the inclusion criterion of being able to sit unsupported has probably influenced this outcome. Furthermore, the inclusion criterion of the ability to follow test instructions may have excluded children with CP who exhibited severe sensory and cognitive impairments. These criteria have led to underrepresentation of children with more severe motor involvement (GMFCS levels IV and V), as well as sensorial and cognitive impairments. This may restrict the generalizability of our findings to the most severely affected segments of the CP population [63]. Finally, the present study did not include variables such as family socioeconomic status, parental stress and environmental helpfulness, which have demonstrated a significant impact on CP-QoL scores [51,52,53,54].

### 4.2. Future Research Directions

Future studies should aim to incorporate self-reported QoL measures for older children and adolescents to capture their unique perspectives, complementing the parent-proxy data obtained in this study. Additionally, expanding the research to include larger cohorts with higher GMFCS levels (IV and V) is essential to provide a more comprehensive understanding of well-being across the full spectrum of CP severity. Furthermore, longitudinal designs are needed to track how the relationship between functional performance and quality of life evolves during critical developmental transitions. Considering socioeconomic status, parental stress, and environmental helpfulness provided a deeper understanding of how these factors interrelate with others affecting the QoL of children with CP and their families. Finally, investigating the impact of family-centered intervention models that prioritize the quality and integration of therapy into natural environments—rather than solely increasing frequency—could provide valuable insights into mitigating treatment burden and enhancing overall family health.

## 5. Conclusions

The findings of this study underscore that QoL in children with CP is a multidimensional construct predominantly driven by functional independence and social participation. Specifically: manual ability (MACS) emerged as a more consistent predictor of social and emotional well-being than gross motor function, highlighting the critical role of hand function in a child’s social integration and self-esteem.

Moreover, functional performance in daily activities (as measured by the PEDI-CAT), rather than mere functional capacity, is the primary determinant of how parents perceive their child’s competence, and this relationship becomes more pronounced as the child ages. Lower levels of functional capacity, as indicated by low GMFM scores and higher functional classification levels (GMCS, MACS, EDACS and CFC) were also associated with a lower QoL.

A high frequency of physical therapy (>2 h per week) was associated with lower parental scores in emotional and family domains. Children who wore AFOs, used wheelchair, had received toxin botulinum injection and/or underwent pelvic surgery also reflected a greater impact on their QoL, suggesting that medicalization has a significant impact on various dimensions of CP-QoL.

To improve QoL in children with CP, all sociodemographic and clinical factors must be evaluated, taking parental opinion into account. The interrelation of all these factors, together with the environmental context, could provide a more significant analysis and enable a more comprehensive rehabilitation approach through models that prioritize activity and participation within natural environments. Clinicians can balance therapeutic goals with preserving family routines and the child’s emotional well-being to achieve a truly holistic improvement in QoL.

## Figures and Tables

**Table 1:** Sociodemographic, clinical, and therapeutic characteristics of children and adolescents with cerebral palsy included in the study.

Variables	n (%)
Age	
5–12 years	46 (48.4)
13–19 years	49 (51.6)
Diagnostic	
Hemiparesis	37 (39.0)
Spastic diplegic CP	32 (33.7)
Spastic quadriplegic CP	20 (21.1)
Ataxic CP	6 (6.3)
Ankle–foot orthoses (AFO)	
None	19 (20.0)
Daytime AFO	48 (50.5)
Nighttime AFO	11 (11.6)
Daytime AFO + Nighttime AFO	17 (17.9)
Assistive devices	
None	70 (73.7)
Walker	2 (2.1)
Wheelchair	5 (5.3)
Walker + Wheelchair	18 (19.0)
Standing frame (Yes)	14 (14.7)
Glasses (Yes)	42 (44.2)
Surgery affecting pelvis (Yes)	21 (22.1)
Botulinum toxin (Yes)	25 (26.3)
Physical therapy	
None or <1 h/week	15 (15.8)
1–2 h/week	32 (33.7)
>2–3 h/week	23 (24.2)
>3 h/week	25 (26.3)
Occupational therapy (>2 h/week)	20 (21.1)
Speech therapy (>2 h/week)	17 (17.9)
School	
Special	6 (6.3)
Ordinary with adaptations	64 (67.4)
Ordinary	25 (26.3)
Sport practice (Yes)	47 (49.5)
Significant cognitive or language disability	23 (24.2)
Father’s educational level	
Primary/Secondary	23 (24.2)
Intermediate	38 (40.0)
University	34 (35.8)
Mother’s educational level	
Primary/Secondary	23 (24.2)
Intermediate	28 (29.5)
University	44 (46.3)

AFO: Ankle–Foot Orthosis; CP: cerebral palsy.

**Table 2:** Functional classification of participants according to GMFCS, MACS, EDACS, and CFCS levels.

	GMFCS	MACS	EDACS	CFCS
I	36 (37.9)	34 (35.8)	69 (72.6)	72 (75.8)
II	39 (41.0)	41 (43.1)	17 (17.9)	13 (13.7)
III	13 (13.7)	15 (15.8)	6 (6.3)	6 (6.3)
IV	7 (7.4)	5 (5.3)	3 (3.2)	4 (4.2)

Data are shown as n (%). GMFCS: Gross Motor Function Classification System; MACS: Manual Ability Classification System; EDACS: Eating and Drinking Ability Classification System; CFCS: Communication Function Classification System.

**Table 3:** Differences in four CPQoL domains according to sociodemographic and therapeutic dichotomous variables.

	Social Well-Being, Acceptance, and Participation			Feelings About Functioning			Emotional Wellbeing and Self-Esteem			Family Health		
	NO	YES	*p*	NO	YES	*p*	NO	YES	*p*	NO	YES	*p*
Ankle–foot orthoses (AFO)	16.0 (15.0–17.0)	14.0 (12.0–16.0)	**0.008**	14.0 (12.0–14.5)	12.0 (11.0–13.3)	**0.035**	14.0 (13.0–15.0)	13.0 (11.0–15.0)	0.103	13.0 (11.4–14.0)	11.0 (10.0–12.0)	**0.003**
Botulinum toxin	15.0 (13.0–17.0)	13.0 (11.0–14.0)	**0.004**	13.0 (11.0–14.0)	13.0 (11.0–14.0)	0.905	14.0 (12.0–15.0)	13.0 (11.0–13.0)	**0.014**	11.5 (10.0–13.0)	10.0 (9.0–12.0)	**0.008**
Surgery affecting pelvis	15.0 (12.3–17.0)	13.0 (12.0–15.0)	0.098	13.0 (11.0–14.0)	12.0 (10.0–14.0)	0.095	14.0 (12.0–15.0)	12.0 (11.0–14.0)	**0.008**	11.0 (10.0–13.0)	11.0 (9.0–12.0)	**0.035**
	<2 h/week	>2 h/week	*p*	<2 h/week	>2 h/week	*p*	<2 h/week	>2 h/week	*p*	<2 h/week	>2 h/week	*p*
Physical therapy	15.0 (13.0–17.0)	14.0 (11.0–16.0)	0.108	14.0 (12.0–14.0)	12.0 (10.0–13.0)	**<0.001**	14.0 (13.0–15.0)	13.0 (11.0–15.0)	**0.027**	12.0 (10.5–13.5)	11.0 (10.0–12.0)	**0.018**
Occupational therapy	15.0 (12.0–16.5)	14.0 (12.8–15.0)	0.337	13.0 (11.0–14.0)	12.0 (11.0–13.0)	**0.038**	14.0 (11.5–15.0)	13.0 (11.8–16.0)	0.825	11.0 (10.0–13.0)	11.5 (10.0–12.0)	0.875

Significant values are in bold. AFO: Ankle–Foot Orthosis.

**Table 4:** Correlations between CPQoL domains and functional-related variables.

	Social Well-Being, Acceptance, and Participation	Feelings About Functioning	Emotional Well-Being and Self-Esteem	Pain and Impact of Disability	School	Access to Services	Family Health
Total GMFM-88	0.239, ***p*** **= 0.020**	0.616, ***p*** **< 0.001**	0.298, ***p*** **= 0.003**	−0.085, *p* = 0.413	0.110, *p* = 0.289	0.292, ***p*** **= 0.004**	0.245, ***p*** **= 0.017**
PEDI-CAT Activity	0.357, ***p*** **< 0.001**	0.738, ***p*** **< 0.001**	0.288, ***p*** **= 0.005**	0.051, *p* = 0.621	0.221, ***p*** **= 0.031**	0.215, ***p*** **= 0.036**	0.245, ***p*** **= 0.017**
PEDI-CAT Mobility	0.266, ***p*** **= 0.009**	0.619, ***p*** **< 0.001**	0.300, ***p*** **= 0.003**	0.090, *p* = 0.388	0.123, *p* = 0.234	0.283, ***p*** **= 0.005**	0.269, ***p*** **= 0.008**
PEDI-CAT Social/Cognitive	0.206, ***p*** **= 0.004**	0.505, ***p*** **< 0.001**	0.080, *p* = 0.440	−0.014, *p* = 0.893	0.255, ***p*** **= 0.013**	−0.001, *p* = 0.993	0.235, ***p*** **= 0.022**
GMFCS	−0.294, ***p*** **= 0.004**	−0.612, ***p*** **< 0.001**	−0.333, ***p*** **< 0.001**	0.003, *p* = 0.980	−0.169, *p* = 0.101	−0.278, ***p*** **= 0.006**	−0.268, ***p*** **= 0.009**
MACS	−0.442, ***p*** **< 0.001**	−0.571, ***p*** **< 0.001**	−0.322, ***p*** **= 0.001**	−0.032, *p* = 0.756	−0.308, ***p*** **= 0.002**	−0.179, *p* = 0.082	−0.294, ***p*** **= 0.004**
EDACS	−0.291, ***p*** **= 0.004**	−0.479, ***p*** **< 0.001**	−0.196, *p* = 0.056	−0.085, *p* = 0.412	−0.076, *p* = 0.467	−0.149, *p* = 0.148	−0.169, *p* = 0.101
CFCS	−0.294, ***p*** **= 0.004**	−0.448, ***p*** **< 0.001**	−0.188, *p* = 0.067	−0.056, *p* = 0.592	−0.075, *p* = 0.470	−0.212, ***p*** **= 0.039**	−0.144, *p* = 0.164
Ankle–foot orthoses (AFO)	−0.117, *p* = 0.259	−0.093, *p* = 0.368	−0.145, *p* = 0.162	−0.007, *p* = 0.943	−0.148, *p* = 0.151	−0.176, *p* = 0.088	−0.132, *p* = 0.202
Walker/wheelchair	−0.189, *p* = 0.067	−0.571, ***p*** **< 0.001**	−0.159, *p* = 0.124	0.040, *p* = 0.703	−0.146, *p* = 0.157	−0.177, *p* = 0.085	−0.207, ***p*** **= 0.045**
Physical Therapy	−0.181, *p* = 0.080	−0.430, ***p*** **< 0.001**	−0.270, ***p*** **= 0.008**	0.044, *p* = 0.669	−0.043, *p* = 0.680	−0.229, ***p*** **= 0.026**	−0.228, ***p*** **= 0.026**
Mother’s education level	−0.085, *p* = 0.414	−0.101, *p* = 0.331	0.010, *p* = 0.924	−0.116, *p* = 0.264	0.069, *p* = 0.503	0.000, *p* = 0.999	0.127, *p* = 0.219
Father’s education level	−0.034, *p* = 0.743	−0.000, *p* = 0.997	−0.034, *p* = 0.742	0.023, *p* = 0.827	−0.074, *p* = 0.475	0.064, *p* = 0.538	0.066, *p* = 0.526

Significant values are in bold. GMFM-88: Gross Motor Function Measure (88 items); GMFCS: Gross Motor Function Classification System; MACS: Manual Ability Classification System; EDACS: Eating and Drinking Ability Classification System; CFCS: Communication Function Classification System.

**Table 5:** Multiple regressions of five of the CPQoL dimensions.

	Adjusted R^2^	Variables (Final Model)	Beta (95% CI)	*p*
Social Well-being, Acceptance, and Participation	0.191, *p* < 0.001	MACS	−1.35 (−1.90, −0.791)	<0.001
Feelings about Functioning	0.619, *p* < 0.001	Total GMFM-88	0.018 (0.012, 0.025)	<0.001
		PEDI-CAT Activity	0.1436 (0.069, 0.218)	<0.001
		PEDI-CAT Social/Cognitive	0.1005 (0.025, 0.176)	0.010
Emotional Well-being and Self-Esteem	0.151, *p* < 0.001	MACS	−0.746 (−1.272, −0.220)	0.006
		Physical therapy	−0.469 (−0.898, −0.041)	0.032
Access to services	0.066, *p* = 0.007	PEDI-CAT Mobility	0.113 (0.032, 0.194)	0.007
Family health	0.0679, *p* = 0.006	MACS	−0.664 (−1.13, −0.193)	0.006

GMFM-88: Gross Motor Function Measure (88 items); MACS: Manual Ability Classification System.

## Data Availability

The data presented in this study are available on request from the corresponding author. The data are not publicly available due to ethical restrictions. For additional information please refer to: javier.lopez3@universidadeuropea.es.

## References

[1] Rosenbaum P., Paneth N., Leviton A., Goldstein M., Bax M., Damiano D., Dan B., Jacobsson B. (2007). A Report: The Definition and Classification of Cerebral Palsy April 2006. Dev. Med. Child Neurol. Suppl..

[2] McIntyre S., Goldsmith S., Webb A., Ehlinger V., Hollung S.J., McConnell K., Arnaud C., Smithers-Sheedy H., Oskoui M., Khandaker G. (2022). Global Prevalence of Cerebral Palsy: A Systematic Analysis. Dev. Med. Child Neurol..

[3] Tan S.S., van Meeteren J., Ketelaar M., Schuengel C., Reinders-Messelink H.A., Raat H., Dallmeijer A.J., Roebroeck M.E. (2014). Long-Term Trajectories of Health-Related Quality of Life in Individuals with Cerebral Palsy: A Multicenter Longitudinal Study. Arch. Phys. Med. Rehabil..

[4] Group W. (1994). Development of the WHOQOL: Rationale and Current Status. Int. J. Ment. Health.

[5] Davis E., Reddihough D., Murphy N., Epstein A., Reid S.M., Whitehouse A., Williams K., Leonard H., Downs J. (2017). Exploring Quality of Life of Children with Cerebral Palsy and Intellectual Disability: What Are the Important Domains of Life?. Child Care Health Dev..

[6] Tsoi W.S.E., Zhang L.A., Wang W.Y., Tsang K.L., Lo S.K. (2012). Improving Quality of Life of Children with Cerebral Palsy: A Systematic Review of Clinical Trials. Child Care Health Dev..

[7] Varni J.W., Seid M., Kurtin P.S. (2001). PedsQLTM 4.0: Reliability and Validity of the Pediatric Quality of Life InventoryTM Version 4.0 Generic Core Scales in Healthy and Patient Populations. Med. Care.

[8] Ravens-Sieberer U., Herdman M., Devine J., Otto C., Bullinger M., Rose M., Klasen F. (2014). The European KIDSCREEN Approach to Measure Quality of Life and Well-Being in Children: Development, Current Application, and Future Advances. Qual. Life Res..

[9] Waters E., Davis E., Mackinnon A., Boyd R., Graham H.K., Kai Lo S., Wolfe R., Stevenson R., Bjornson K., Blair E. (2007). Psychometric Properties of the Quality of Life Questionnaire for Children with CP. Dev. Med. Child Neurol..

[10] Davis E., Mackinnon A., Davern M., Boyd R., Waters E., Graham H.K., Reid S., Reddihough D. (2013). Description and Psychometric Properties of the CP QOL-Teen: A Quality of Life Questionnaire for Adolescents with Cerebral Palsy. Res. Dev. Disabil..

[11] Badia M., Orgaz M.B., Riquelme I., Gómez-Iruretagoyena J. (2020). Domains of the Cerebral Palsy Quality of Life Questionnaire (CP QOL) for Children and Adolescents: Spanish Adaptation and Psychometric Properties. J. Dev. Phys. Disabil..

[12] McDougall J., Wright V., Schmidt J., Miller L., Lowry K. (2011). Applying the ICF Framework to Study Changes in Quality-of-Life for Youth with Chronic Conditions. Dev. Neurorehabilit..

[13] Findlay B., Switzer L., Narayanan U., Chen S., Fehlings D. (2016). Investigating the Impact of Pain, Age, Gross Motor Function Classification System, and Sex on Health-related Quality of Life in Children with Cerebral Palsy. Dev. Med. Child Neurol..

[14] Nurani Gharaborghe S., Sarhady M., Hosseini S.M.S., Mortazavi S.S. (2015). Quality of Life and Gross Motor Function in Children with Cerebral Palsy (Aged 4–12). Iran. Rehabil. J..

[15] Davis E., Young D., Gilson K.M., Swift E., Chan J., Gibbs L., Tonmukayakul U., Reddihough D., Williams K. (2018). A Rights-Based Approach for Service Providers to Measure the Quality of Life of Children with a Disability. Value Health.

[16] Dickinson H.O., Parkinson K.N., Ravens-Sieberer U., Schirripa G., Thyen U., Arnaud C., Beckung E., Fauconnier J., McManus V., Michelsen S.I. (2007). Self-Reported Quality of Life of 8–12-Year-Old Children with Cerebral Palsy: A Cross-Sectional European Study. Lancet.

[17] Davis E., Waters E., Mackinnon A., Reddihough D., Boyd R., Graham H.K. (2008). Quality of Life of Children with CP: Condition-specific Instrument and Proxy Reports. Dev. Med. Child Neurol..

[18] Carlberg E.B., Hadders-Algra M. (2005). Postural Dysfunction in Children with Cerebral Palsy: Some Implications for Therapeutic Guidance. Neural Plast..

[19] Chakraborty S., Nandy A., Kesar T.M. (2020). Gait Deficits and Dynamic Stability in Children and Adolescents with Cerebral Palsy: A Systematic Review and Meta-Analysis. Clin. Biomech..

[20] Ko J., Kim M. (2013). Reliability and Responsiveness of the Gross Motor Function Measure-88 in Children with Cerebral Palsy. Phys. Ther..

[21] Ferre-Fernández M., Murcia-González M.A., Ríos-Díaz J. (2022). Intra- and Interrater Reliability of the Spanish Version of the Gross Motor Function Measure (GMFM-SP-88). Pediatr. Phys. Ther..

[22] Palisano R.J., Rosenbaum P., Bartlett D., Livingston M.H. (2008). Content Validity of the Expanded and Revised Gross Motor Function Classification System. Dev. Med. Child Neurol..

[23] Eliasson A.C., Krumlinde-Sundholm L., Rösblad B., Beckung E., Arner M., Öhrvall A.M., Rosenbaum P. (2006). The Manual Ability Classification System (MACS) for Children with Cerebral Palsy: Scale Development and Evidence of Validity and Reliability. Dev. Med. Child Neurol..

[24] Hidecker M.J.C., Paneth N., Rosenbaum P.L., Kent R.D., Lillie J., Eulenberg J.B., Cherster K., Johnson B., Michalsen L., Evatt M. (2011). Developing and Validating the Communication Function Classification System for Individuals with Cerebral Palsy. Dev. Med. Child Neurol..

[25] Sellers D., Mandy A., Pennington L., Hankins M., Morris C. (2014). Development and Reliability of a System to Classify the Eating and Drinking Ability of People with Cerebral Palsy. Dev. Med. Child Neurol..

[26] Morris C., Kurinczuk J.J., Fitzpatrick R., Rosenbaum P.L. (2006). Do the Abilities of Children with Cerebral Palsy Explain Their Activities and Participation?. Dev. Med. Child Neurol..

[27] Dumas H.M., Fragala-Pinkham M.A., Rosen E.L., Lombard K.A., Farrell C. (2015). Pediatric Evaluation of Disability Inventory Computer Adaptive Test (PEDI-CAT) and Alberta Infant Motor Scale (AIMS): Validity and Responsiveness. Phys. Ther..

[28] World Health Organization (2007). International Classification of Functioning, Disability, and Health: Children & Youth Version: ICF-CY.

[29] Jackman M., Sakzewski L., Morgan C., Boyd R.N., Brennan S.E., Langdon K., Toovey R.A.M., Greaves S., Thorley M., Novak I. (2022). Interventions to Improve Physical Function for Children and Young People with Cerebral Palsy: International Clinical Practice Guideline. Dev. Med. Child Neurol..

[30] López-Ruiz J., Estrada-Barranco C., Martín-Gómez C., Egea-Gámez R.M., Valera-Calero J.A., Martín-Casas P., López-de-Uralde-Villanueva I. (2023). Trunk Control Measurement Scale (TCMS): Psychometric Properties of Cross-Cultural Adaptation and Validation of the Spanish Version. Int. J. Environ. Res. Public Health.

[31] López-Ruiz J., Estrada-Barranco C., Giménez-Mestre M.J., Villarroya-Mateos I., Martín-Casas P., López-de-Uralde-Villanueva I. (2023). Differences between Novice and Expert Raters Assessing Trunk Control Using the Trunk Control Measurement Scale Spanish Version (TCMS-S) in Children with Cerebral Palsy. J. Clin. Med..

[32] Cuschieri S. (2019). The STROBE Guidelines. Saudi J. Anaesth..

[33] Longo E., Badia M., Orgaz M.B., Gómez-vela M. (2017). Comparing Parent and Child Reports of Health-Related Quality of Life and Their Relationship with Leisure Participation in Children and Adolescents with Cerebral Palsy. Res. Dev. Disabil..

[34] Almasri N.A., Alquaqzeh F.A. (2023). Determinants of Quality of Life of Children and Adolescents with Cerebral Palsy: A Systematic Review. Phys. Occup. Ther. Pediatr..

[35] Aza A., Riquelme I., Gómez Vela M., Badia M. (2024). Proxy- and Self-Report Evaluation of Quality of Life in Cerebral Palsy: Using Spanish Version of CPQOL for Children and Adolescents. Res. Dev. Disabil..

[36] Mancini M.C., Coster W.J., Amaral M.F., Avelar B.S., Freitas R., Sampaio R.F. (2016). New Version of the Pediatric Evaluation of Disability Inventory (PEDI-CAT): Translation, Cultural Adaptation to Brazil and Analyses of Psychometric Properties. Braz. J. Phys. Ther..

[37] Haley S.M., Coster W.J., Dumas H.M., Fragala-Pinkham M.A., Kramer J., Ni P., Tian F., Kao Y.C., Moed R., Ludlow L.H. (2011). Accuracy and Precision of the Pediatric Evaluation of Disability Inventory Computer-Adaptive Tests (PEDI-CAT). Dev. Med. Child Neurol..

[38] PEDI CAT—Pearson Clinical Assessment España. https://www.pearsonclinical.es/pedi-cat?utm_source=google&utm_medium=cpc&utm_campaign=16142275884&utm_content=159106567625&utm_term=pedi%20cat&utm_source=google&utm_medium=cpc&utm_campaign=OT_products&utm_content=PEDI-CAT&utm_id=159106567625&gad_source=1&gad_campaignid=16142275884&gbraid=0AAAAADyRHBqxGnNZA5dB-k9NB5keTxhGQ&gclid=Cj0KCQiApfjKBhC0ARIsAMiR_Itfe_LfFP5yWJPvDiRux3e97TowfviuA5jjQscTZLBLzeJTikImJecaAhIwEALw_wcB.

[39] Russell D.J., Hart H. (2013). Gross motor function measure (GMFM-66 & GMFM-88) User’s manual. Clinics in Developmental Medicine.

[40] Alotaibi M., Long T., Kennedy E., Bavishi S. (2013). The Efficacy of GMFM-88 and GMFM-66 to Detect Changes in Gross Motor Function in Children with Cerebral Palsy (CP): A Literature Review. Disabil. Rehabil..

[41] Ferre-Fernández M., Murcia-González M.A., Ríos-Díaz J. (2020). Translation and Cross-Cultural Adaptation of the Gross Motor Function Measure to the Spanish Population of Children with Cerebral Palsy. Rev. Neurol..

[42] Palisano R.J., Rosenbaum P.L., Walter S.D., Russell D.J., Wood E., Galuppi B.E. (1997). Development and Reliability of a System to Classify Gross Motor Function in Children with Cerebral Palsy. Class. Pap. Orthop..

[43] Russell D., Avery L., Rosenbaum P., Raina P., Walter S., Palisano R. (2000). Improved Scaling of the Gross Motor Function Measure for Children with Cerebral Palsy: Evidence of Reliability and Validity. Phys. Ther..

[44] Palisano R.J., Avery L., Gorter J.W., Galuppi B., McCoy S.W. (2018). Stability of the Gross Motor Function Classification System, Manual Ability Classification System, and Communication Function Classification System. Dev. Med. Child Neurol..

[45] Mukaka M.M. (2012). Statistics Corner: A Guide to Appropriate Use of Correlation Coefficient in Medical Research. Malawi. Med. J..

[46] Carnahan K.D., Arner M., Hägglund G. (2007). Association between Gross Motor Function (GMFCS) and Manual Ability (MACS) in Children with Cerebral Palsy. A Population-Based Study of 359 Children. BMC Musculoskelet. Disord..

[47] Gunel M.K., Mutlu A., Tarsuslu T., Livanelioglu A. (2009). Relationship among the Manual Ability Classification System (MACS), the Gross Motor Function Classification System (GMFCS), and the Functional Status (WeeFIM) in Children with Spastic Cerebral Palsy. Eur. J. Pediatr..

[48] Compagnone E., Maniglio J., Camposeo S., Vespino T., Losito L., De Rinaldis M., Gennaro L., Trabacca A. (2014). Functional Classifications for Cerebral Palsy: Correlations between the Gross Motor Function Classification System (GMFCS), the Manual Ability Classification System (MACS) and the Communication Function Classification System (CFCS). Res. Dev. Disabil..

[49] Elema A., Zalmstra T.A.L., Boonstra A.M., Narayanan U.G., Reinders-Messelink H.A., Annette A.A.J. (2016). Pain and Hospital Admissions Are Important Factors Associated with Quality of Life in Nonambulatory Children. Acta Paediatr. Int. J. Paediatr..

[50] Power R., Galea C., Muhit M., Heanoy E., Karim T., Badawi N., Khandaker G. (2020). What Predicts the Proxy-Reported Health-Related Quality of Life of Adolescents with Cerebral Palsy in Bangladesh?. BMC Public Health.

[51] Blasco M., García-Galant M., Laporta-Hoyos O., Ballester-Plané J., Jorba-Bertran A., Caldú X., Miralbell J., Alonso X., Meléndez-Plumed M., Toro-Tamargo E. (2023). Factors Related to Quality of Life in Children with Cerebral Palsy. Pediatr. Neurol..

[52] Di Lieto M.C., Matteucci E., Martinelli A., Beani E., Menici V., Martini G., Barzacchi V., Dubbini N., Sgandurra G. (2025). Impact of Social Participation, Motor, and Cognitive Functioning on Quality of Life in Children with Cerebral Palsy. Res. Dev. Disabil..

[53] Chen K.L., Tseng M.H., Shieh J.Y., Lu L., Huang C.Y. (2014). Determinants of Quality of Life in Children with Cerebral Palsy: A Comprehensive Biopsychosocial Approach. Res. Dev. Disabil..

[54] Gelkop N., Engel-Yeger B. (2025). Participation, Environment, and Quality of Life in Children with Cerebral Palsy and Physical Disabilities. Dev. Med. Child Neurol..

[55] Waters E., Shelly A., Davis E. (2011). Condition-Specific Instruments to Measure the Quality of Life (QoL) of Children and Adolescents with Cerebral Palsy (CP). Quality of Life Measurement in Neurodegenerative and Related Conditions.

[56] Keawutan P., Bell K.L., Oftedal S., Davies P.S.W., Ware R.S., Boyd R.N. (2018). Quality of Life and Habitual Physical Activity in Children with Cerebral Palsy Aged 5 Years: A Cross-Sectional Study. Res. Dev. Disabil..

[57] Sharawat I.K., Panda P.K. (2022). Quality of Life and Its Association with Level of Functioning in Young Children with Cerebral Palsy. Neuropediatrics.

[58] Badia M., Riquelme I., Orgaz B., Acevedo R., Longo E., Montoya P. (2014). Pain, Motor Function and Health-Related Quality of Life in Children with Cerebral Palsy as Reported by Their Physiotherapists. BMC Pediatr..

[59] Rapp M., Eisemann N., Arnaud C., Ehlinger V., Fauconnier J., Marcelli M., Michelsen S.I., Nystrand M., Colver A., Thyen U. (2017). Predictors of Parent-Reported Quality of Life of Adolescents with Cerebral Palsy: A Longitudinal Study. Res. Dev. Disabil..

[60] Shore B.J., Allar B.G., Miller P.E., Matheney T.H., Snyder B.D., Fragala-Pinkham M. (2019). Measuring the Reliability and Construct Validity of the Pediatric Evaluation of Disability Inventory–Computer Adaptive Test (PEDI-CAT) in Children With Cerebral Palsy. Arch. Phys. Med. Rehabil..

[61] Shelly A., Davis E., Waters E., Mackinnon A., Reddihough D., Boyd R., Reid S., Graham H.K. (2008). The Relationship between Quality of Life and Functioning for Children with Cerebral Palsy. Dev. Med. Child Neurol..

[62] Milićević M. (2023). Functional and Environmental Predictors of Health-related Quality of Life of School-age Children with Cerebral Palsy: A Cross-sectional Study of Caregiver Perspectives. Child Care Health Dev..

[63] Makris T., Dorstyn D., Crettenden A. (2021). Quality of Life in Children and Adolescents with Cerebral Palsy: A Systematic Review with Meta-Analysis. Disabil. Rehabil..

